# Replantation of multiple fingertip amputations using super microsurgery: A case report and literature review

**DOI:** 10.1016/j.jpra.2024.03.008

**Published:** 2024-03-28

**Authors:** Zhegang Zhou, Longbiao Yu, Fanbin Meng, Jingjing Wen, Yingfeng Xiao, Bo Zhou, Shengxiang Wan, Hui Zeng, Fei Yu

**Affiliations:** a Department of Hand & Microsurgery, Peking University Shenzhen Hospital, China; b Department of Orthopedics, Shenzhen Second People's Hospital, China; c Department of Bone & Joint Surgery, Peking University Shenzhen Hospital, China; d National & Local Joint Engineering Research Center of Orthopaedic Biomaterials, China; e Shenzhen Key Laboratory of Orthopaedic Diseases and Biomaterials Research, China

**Keywords:** Super microsurgery, Multiple fingertip detachment injuries, Hand function reconstruction, Special medical case

## Abstract

**Background:**

The fingertip amputation is an amputation type of the finger beyond the proximal nail fold. There is no vein available for anastomoses on the dorsal side of the finger, and the palmar vein of the finger is small and tightly attached to the skin. Therefore, it is relatively difficult to implement surgical anastomoses, which poses challenges to the clinical treatment of fingertip amputations.

**Case report:**

A 29-year-old male was admitted to the hospital due to “the amputation of the fingertips of the right index, middle, and ring fingers caused by a heavy object compression 3 h ago”. The admission examination revealed that the right index, middle, and ring fingers were completely severed at the 1/2 plane of the nail bed, with irregular sections, severe contusion, and pollution. The X-ray examination showed comminuted fractures of the distal phalanges of the right index, middle, and ring fingers. Based on these findings, the patient was diagnosed with multiple severed fingertips of the right hand (Tamai Zone 1). The patient underwent debridement, vascular exploration, and replantation of the right index, middle, and ring fingertips under emergency general anesthesia. After surgery, anti-inflammatory, spasmolytic, and anticoagulant treatment and regular dressing changes were conducted. The patient did not receive a blood transfusion, and all three fingers survived. The appearance of these fingers was favorable 3 months after surgery, and the flexion and extension of these fingers were normal. Eventually, the patient achieved excellent Chen's hand function scores.

**Conclusions:**

To the best of our knowledge, this may be the first successful case regarding the replantation of three fingertips after amputations in Tamai Zone 1 with favorable outcomes. It can be maintained that super microsurgery can be used for the replantation of multiple fingertip amputations.

## Introduction

In trauma-induced finger detachment, removal during debridement is required for those without any tissue connection or even if there is an injured or inactivated tissue connection. As one amputation type, fingertip detachment involves an amputation with the plane of finger detachment located far from the proximal nail fold. There is no vein for anastomoses on the dorsal side of the finger, and the palmar vein of the finger is small and close to the subcutaneous tissue, thus posing challenges to surgical anastomoses. Especially, fingertip detachment in Tamai Zone 1 (Tamai classification is the most commonly used classification system for distal phalangeal amputation, mainly including two anatomical zones, with the line passing through the phalanges as the boundary between the two zones) is a clinical treatment difficulty.^1^^,^^2^ Fingertip amputations constitute a common type of finger amputation injury, as the tissue is far from the fracture plane, which often leads to severe contusion and a low success rate of replantation. Further, the function and appearance of the hand may be affected, thus imposing a heavy medical burden on patients and families. In this study, a patient with right-hand index, middle, and ring fingertip detachment injuries in Tamai Zone 1 was admitted and treated in our hospital. Super microsurgery was applied to the replantation of the three fingertips, and satisfactory results were achieved. The function and appearance of these fingers achieved a full recovery 3 months after surgery. This case is reported as follows.

## Case report

**Figure 1:**
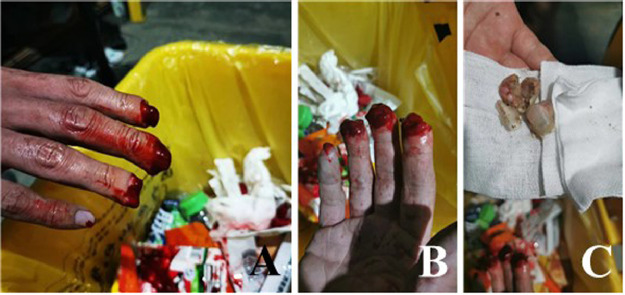
Conditions at admission.

A 29-year-old male was admitted to the hospital due to “the amputation of the fingertips of the right index, middle, and ring fingers caused by a heavy object compression 3 h ago”. The admission examination revealed that the right index, middle, and ring fingers were completely severed at the 1/2 plane of the nail bed, with irregular sections, severe contusion, and pollution. The X-ray examination showed comminuted fractures of the distal phalanges of the right index, middle, and ring fingers. Based on these findings, the patient was diagnosed with multiple severed fingertips in the right hand (Tamai Zone 1) (Figure 1).

**Figure 2:**
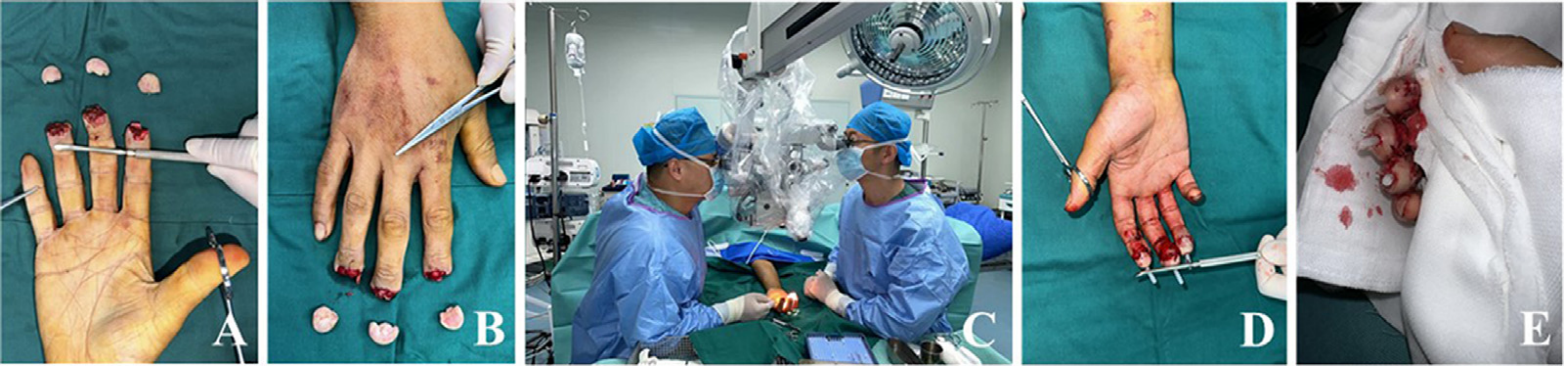
Perioperative and post-operative conditions.

Treatment process: The patient underwent debridement, vascular exploration, and replantation of the right index, middle, and ring fingertips under emergency general anesthesia. During surgery, it was found that the right-hand index, middle, and ring fingers were completely severed at a distance of 1/2 of the nail bed plane. The wound surface was irregular, with severe contusion and contamination. Extensive bruises were observed on the severed finger body. After debridement and observation under the microscope, avulsion injuries were identified. The digital artery was severed at the distal end of the arcuate artery and avulsed 5 mm toward the proximal cardiac segment. Under the microscope, debridement was performed, and the phalanx was shortened by about 3 mm. The fracture was fixed with a Kirschner wire. After the proximal digital artery was detected and the severed end was trimmed by 2 mm, it was found that the blood spurt was good. Through the exploration of the blood vessels in the severed finger body, an arcuate artery was detected at the central position of the finger body on the palmer side. The arcuate artery was found to be avulsed by 0.5 cm from the proximal segment. However, after the severed end was trimmed by 2 mm, the lumen was still acceptable. In the exploration under a 30x microscope, the diameter of the artery was measured to be about 0.5 mm. The end-to-end anastomosis of the arcuate artery was performed using super microsurgery, and 6–8 stitches were applied to the anastomosis under a 30 × microscope. The replantation was performed in the order of the index, middle, and ring fingers, with each finger anastomosed with one artery and no vein. The surgery continued for 5 h (Figure 2). After surgery, the nail bed was bled to eliminate the reflux.

Post-operative treatment: The patient received anti-inflammatory, spasmolytic, and anticoagulant treatment. Antibiotic drugs (1.0 g of cefalexin) were administered twice daily for a total of 3 days; anticoagulant drugs (0.4 ml of low molecular weight heparin) were administered subcutaneously once a day for a total of 5 days. After surgery, there was blood leakage at the nail bed, and regular dressing changes were performed without blood transfusion treatment.

**Figure 3:**
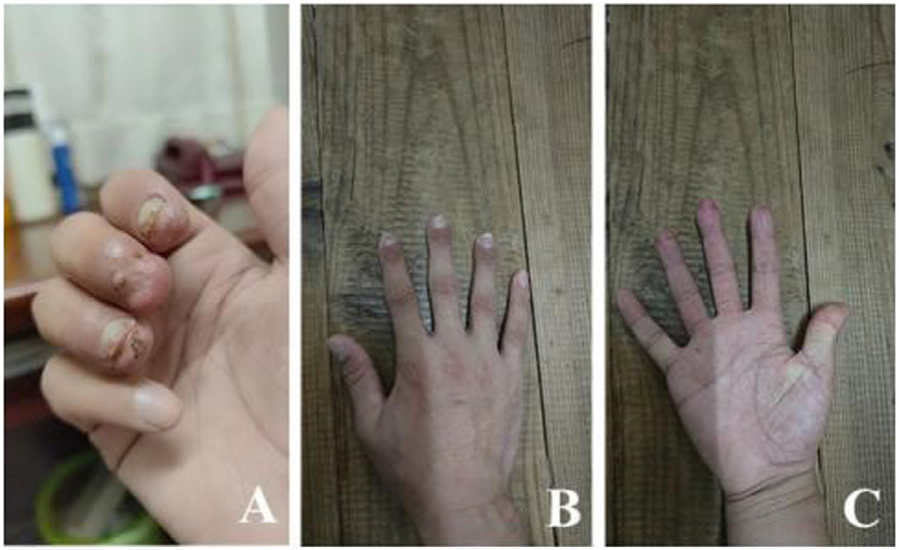
Long-term effects.

Treatment effect: All three fingers of the patient survived after surgery. The appearance of these fingers was favorable 3 months after surgery, and the flexion and extension of these fingers were normal. Eventually, the patient achieved excellent Chen's hand function scores (Figure 3).

## Discussion

Finger amputation injury is a common hand injury.^3^ Since the first successful replantation of severed fingers reported by Chen Zhongwei in 1965, a series of breakthroughs have been made in China. The appearance and function of the hand after replantation are related to the injury severity, replantation repair level, and rehabilitation status. Fingertip detachment injury can be classified as a special type of finger detachment injury, and the difficulty in replantation is significantly increased due to small blood vessels. In this study, the patient underwent replantation of 3 fingertip amputations in Tamai Zone 1 using super microsurgery and achieved favorable therapeutic results. The blood vessels in this area are the smallest, posing enormous challenges to replantation. If the injury mechanism of fingertip detachment is crushing and contusion, the tissue contusion would be severe, which will increase the difficulty of replantation. In this case, the patient suffered from heavy object crushing and avulsion injuries, and the tissue contusion was seriously contaminated. The detachment plane was far from the 1/2 plane of the nail bed, and the arcuate artery was subjected to retrograde avulsion, making the replantation more difficult. The experience in the successful management of this patient is summarized as follows.

### Why did we consider using super microsurgery

(1) Since the first report on super microsurgery by Koshima in 1999, this technique has been widely used in both microsurgery^4^ and lymphatic surgery.^5^ The pipeline anastomosis with a diameter less than 0.8 mm can be classified into the field of super microsurgery, and the diameter of blood vessels in fingertip injuries is often less than 0.8 mm. Super microsurgery is appropriate for the treatment of these injuries. (2) Our team has attempted fingertip replantation multiple times under a regular microscope before using super microsurgery. However, the regular microscope is limited by the low magnification (the maximum magnification of a regular microscope is 16–22 ×), which cannot be used to effectively present the vascular lumen during anastomoses. (3) Before the application of super microsurgery in the past, 10–0 micro sutures were often used. This suture has some limitations in fingertip replantation, such as excessively thick needles, difficulty in suturing, and failure in effective knot and suture. In the previous studies, when conventional methods were used for anastomoses, only 2–3 stitches could be anastomosed at the level of the arcuate artery, and it was difficult to ensure the quality of anastomoses. For that reason, patients may give up replantation midway due to such limitations as microscope magnification and thicker sutures during vascular exploration or anastomosis attempts or become reluctant to receive replantation due to the inability to ensure the quality of anastomoses. Under these circumstances, only a few fingers survive. (4) In this case, super microsurgery demonstrated significant advantages. Our team has used this technique to complete 80 lymphatic vein anastomoses with anastomotic lumens ranging from 0.3 to 0.5 mm before implementing fingertip replantation in this case. Therefore, we could ensure operational technology and quality. The vessel diameter of the arcuate artery is 0.5–0.6 mm, which is suitable for anastomoses by super microsurgery. Therefore, in this case, the lumen of the arcuate artery can be clearly explored under a 30x microscope, and the lumen can be well trimmed with a clear field of view. Then, super microsurgical instruments and 11–0 sutures can be used for better end-to-end anastomoses.

### Why was the vein not anastomosed

In 2018, Ryu et al.^6^ reported that multiple anastomotic veins can reduce post-operative care intensity, but the number of anastomotic veins was not directly related to survival. Matsuda et al.^7^ and Efanov et al.^8^ also reached a similar conclusion, indicating that regardless of the number of venous anastomoses, there was a high survival rate of fingertips in replantation. Cheng et al.^9^ reported that when fingertip injuries were treated using only arterial anastomoses, excluding venous anastomoses, external bleeding would not occur, which may achieve better surgical outcomes than composite transplantation. Multiple reports from other scholars also confirmed that simple arterial anastomoses were sufficient to restore the blood supply in fingertip replantation.^10^, ^11^, ^12^, ^13^

In this study, the patient had an injury plane that was far beyond the arcuate artery, and in some cases, anastomotic veins could be found on the palm or nail bed side of the plane. However, the patient had severe contusion, and no anastomotic veins were found during exploration. Therefore, post-operative nail bed bleeding was used to maintain venous reflux, which proved to be feasible in previous tissue transplantation or fingertip reconstruction cases. The successful treatment of this patient was closely related to nail bed bleeding and continuous anticoagulation after surgery, which also confirmed the possibility of successful fingertip replantation without venous anastomoses.

### Research progress in the treatment of fingertip injuries

In 1977, Elsahy introduced the indications for fingertip replantation and provided guidance on the treatment of related patients.^14^ In 1980, Allen^15^ used conservative methods to treat fingertip injuries. Later, Japanese doctors performed replantation surgery on fingertip injuries using microscopic techniques and achieved favorable outcomes.^16^ To promote advancements in this field, Strauch et al.^17^ depicted the distribution of hand blood vessels through autopsy. Wei et al.^18^ extended the time for finger replantation after injury using cooling technology. Tsai et al.^19^ proposed an open vessel technique to increase the success rate of fingertip replantation. In 1992, Koshima et al.^20^ reported the role of arteriovenous anastomoses in the replantation of fingertip injuries. In the same year, Foucher et al.^21^ reported the clinical efficacy of distal fingertip replantation and achieved favorable outcomes. In 2001, Hattori et al.^22^ proposed a technique for inserting a vein graft and anastomosing it with the distal branch of the digital artery and the subcutaneous vein of the amputated thumb fingertip. In 2004, Venkatramani et al.^23^ developed a limb fixation method for fingertip replantation, reducing difficulties in fingertip replantation. In the following year, Sabapathy et al.^24^ proposed the concept of non-fixed distal fingertip replantation, which also achieved bone healing in patients with fingertip injuries; Sabapathy et al.^24^ applied the same concept to achieve successful replantation of fingertip injuries. Hattori et al.^25^ compared the differences between microsurgical replantation and amputation closure in single-finger amputation surgeries. The replantation for a single-finger amputation can minimize pain, enhance functional effectiveness, improve appearance, and increase patient satisfaction. Unless patients would like to resume their work early through simple surgery, they may choose amputation closure surgery. Koshima et al.^26^ used delayed venous anastomoses in the replantation treatment of fingertip injuries, and all fingers undergoing reoperation were successfully drained through additional single or double venous drainage of the venous graft. Lee et al.^27^ proposed the effect of the number and proportion of repaired arteries and veins on the survival rate of fingers in replantation, and indicated that there were differences in the repair of blood vessels among different regions. Shieh et al.^28^ maintained that performing related exercises after the replantation treatment of fingertip injuries can affect the sensory recovery of fingers. Later, with the advancement in technology, super microsurgery, tissue engineering, and other techniques emerged and developed. On that basis, more methods for repairing fingertip injuries emerged, such as the double or single clip intravascular stent flipping technology developed by Tsumura et al.,^29^ which improved the success rate of arteriovenous anastomoses. Moreover, autologous^30^ or allogeneic^31^ transplantation can also achieve functional recovery of damaged fingers.

### Application of super microsurgery in replantation of severed fingertips

Pamuk^32^ retrospectively analyzed the selection of replantation surgery or flap surgery in the Tamai Zone I fingertip amputation. He demonstrated that with the emergence of super microsurgery, these patients should undergo replantation surgery first. Even if the replantation treatment was not successful, the results of secondary recovery were still satisfactory. Braig et al.^33^ retrospectively analyzed the success rate of replantation surgery in 11 fingertip amputations in Tamai Zone I and II using super microsurgery (including 6 cases of total resection, 4 cases of subtotal resection, and 1 case of finger pulp avulsion). Among these 11 patients, 8 patients underwent long-term reattachment of amputated tissues, and 3 patients required secondary amputation closure surgery. In the follow-up, they found that 5 patients were satisfied with the efficacy and supported replantation surgery, and 4 patients achieved satisfactory hand functions, and 2 patients had limited hand functions. They confirmed that super microsurgery can be applied to fingertip replantation. Hayashi et al.^34^ conducted a 10-year follow-up of 34 fingertip replantations in 31 patients and found that patients with fingertip replantation did not experience chronic pain, had satisfactory sensory recovery, and had no difference in volume compared with normal fingers. Further, 97% of patients were satisfied with the surgical results. Tashiro et al.^35^ used super microsurgery to perform lymphatic anastomoses, free perforator flap transfer, and fingertip replantation based on semi-vascular stent implantation. All flaps survived perforator-perforator anastomoses and fingertip replantation through super microsurgery. Koshima et al.^36^ described three patients successfully receiving fingertip replantation using super microsurgery. Their team used this technique to perform terminal branch anastomoses of the digital artery. Among the 16 fingers in the three cases, 7 fingers experienced post-operative venous congestion, and 5 fingers underwent delayed venous drainage under the finger block before re-surgery (all fingers were successfully drained through additional single or double venous drainage of the venous graft), resulting in the survival of 13 fingers. Although there are few reports on the use of super microsurgery in the replantation of severed fingertips, the treatment results are good and worthy of promotion. However, previous scholars have not proposed the significance of increasing microscope magnification and upgrading sutures for small vessel anastomoses in fingertip replantation. As mentioned earlier, these two points should also be emphasized in the application of super microsurgery in fingertip replantation.

In this study, this male patient underwent the same post-operative treatment as conventional finger replantation treatment, receiving anti-infection and anticoagulant treatment. He was discharged 5 days after surgery. The patient began finger flexion and extension exercises 2 weeks after surgery. The blood scab wrapping of the post-operative wound was not treated temporarily, the dressing and suture were changed, and the Kirschner wire was removed one month after the surgery. During the follow-up 3 months after surgery, the function and appearance of the hand were favorable. Through the treatment of this patient, it can be maintained that the long-term function and appearance of the hand can achieve an optimal recovery after successful replantation despite difficulties in fingertip replantation. Such patients should actively strive for fingertip replantation.

In conclusion, to the best of our knowledge, this may be the first successful case regarding simultaneous replantation of three fingertips in Tamai Zone 1 using super microsurgery, achieving satisfactory hand functions and appearance in the long term. This broadens the indications for fingertip replantation and provides ideas for the application of super microsurgery in fields other than lymphedema and perforator flaps.

## Authors’ note

The authors have read the CARE Checklist (2016), and the manuscript was prepared and revised according to the CARE Checklist (2016).

## Data availability statement

The raw data supporting the conclusions of this article will be made available by the authors, without undue reservation.

## Ethics statement

Not applicable. Written informed consent was obtained from the participant/s for the publication of this case report.

## Patient consent statement

Written informed consent was obtained from the individual(s) for the publication of any potentially identifiable images or data included in this article.

## CRediT authorship contribution statement

**Zhegang Zhou:** Conceptualization, Investigation, Validation, Writing – original draft. **Longbiao Yu:** Investigation, Writing – original draft. **Fanbin Meng:** Investigation. **Jingjing Wen:** Investigation, Writing – original draft. **Yingfeng Xiao:** Supervision. **Bo Zhou:** Investigation. **Shengxiang Wan:** Supervision. **Hui Zeng:** Conceptualization, Funding acquisition, Supervision, Validation. **Fei Yu:** Conceptualization, Funding acquisition, Supervision, Validation, Writing – review & editing.

## Declaration of competing interest

The data and results of this study can be obtained from the corresponding author according to reasonable requirements. There is no conflict of interest between the authors.
